# West Nile Virus in Morocco, 2003

**DOI:** 10.3201/eid1102.040817

**Published:** 2005-02

**Authors:** Isabelle Schuffenecker, Christophe N. Peyrefitte, Mohammed el Harrak, Séverine Murri, Agnès Leblond, Hervé G. Zeller

**Affiliations:** * Institut Pasteur, Lyon, France;; † Institut de Médecine Tropicale du Service de Santé des Armées, Marseille, France;; ‡ Laboratoire Bio-Pharma, Rabat, Morocco;; § Ecole Vétérinaire de Lyon, Marcy l'Etoile, France

**Keywords:** dispatch, West Nile, Morocco, phylogenetic analysis, equine encephalitis

## Abstract

West Nile virus (WNV) reemerged in Morocco in September 2003, causing an equine outbreak. A WNV strain isolated from a brain biopsy was completely sequenced. On the basis of phylogenetic analyses, Moroccan WNV strains isolated during the 1996 and 2003 outbreaks were closely related to other strains responsible for equine outbreaks in the western Mediterranean basin.

In the early 1950s, scientists first recognized that West Nile virus (WNV) reached outbreak levels in humans in Egypt and Israel (1,2). Initially considered a minor arbovirus, WNV has recently emerged as a major public health and veterinary concern in southern Europe, the Mediterranean basin, and the United States and Canada (1–3). Several outbreaks of severe human meningoencephalitis with fatalities have been reported within the last 8 years in Europe and North Africa, specifically in Romania (1996), Russia (1999), Israel (2000), and Tunisia (1997, 2003) (1,3). Epizootics in horses have also been documented in Morocco (1996), Italy (1998), France (2000), and Israel (2000) (1,4). WNV was responsible for a cluster of human and equine cases in southern France in 2003 (5,6).

On the basis of phylogenetic analyses, WNV strains isolated since 1996 in southern Europe and the Mediterranean basin belong to the clade 1a of lineage 1 (7,8). Moreover, these strains belong to 2 distinct genotypes (8,9). One cluster includes equine strains isolated in Italy and France, human strains isolated in Russia and Israel, and mosquito strains isolated in Romania and Kenya. The other cluster includes most of the strains isolated from birds and horses in Israel from 1997 to 2001 and the North American isolates. Only 5 strains isolated in the Mediterranean basin have been completely sequenced.

Since the first WNV outbreak in Morocco in 1996, which caused 94 cases in equines (including 42 deaths) and 1 case in a human (10), no WNV infections have been reported. An outbreak of WNV occurred among horses stabled in the Moroccan province of Kenitra in September and October 2003. The complete genome sequence of a WNV strain isolated from a brain biopsy was characterized, as well as the complete genome sequence of a strain isolated during the Morocco 1996 WNV outbreak. We studied phylogenetic relationships of the 2 Moroccan strains with other WNV strains isolated in the Mediterranean basin.

## The Study

During the fall of 2003, 9 equine WNV cases were reported to the Moroccan Ministry of Agriculture. All horses had acute neurologic symptoms, fever, paresis of the hindquarters, paralysis, or some combination of these symptms (Table); 5 horses were euthanized. Clinical cases occurred from September 12 to October 1, 2003. No abnormal bird deaths were observed, and no human cases were reported.

**Table:** Clinical and epidemiologic features of 9 horses with confirmed West Nile virus (WNV) infection, Kenitra Province, Morocco*

Locality	Age (y)	Sex	Date of symptoms	Clinical data	Death
Ouled Slama	6	M	Sept 12, 2003	Paralysis	–
Ameur Seflia	7	M	Sept 11, 2003	Paralysis, fever	+
?	8	F	Sept 07, 2003	Paralysis	+
Mograne	7	F	Sept 14, 2003	Paralysis	+
Ameur Seflia	6	M	Sept 19, 2003	Ataxia, fever	–
Ameur Seflia	7	M	Sept 23, 2003	Ataxia	–
Mograne	6	M	Sept 29, 2003	Paresis, fever	+
Mograne	10	F	Oct 1, 2003	Paresis, fever	+†
Ameur Seflia	10	F	?	Paresis, fever	–

*(y), years of age; M, male; F, female. †Source of the Morocco 2003 WNV strain (04.05).

**Figure 1:**
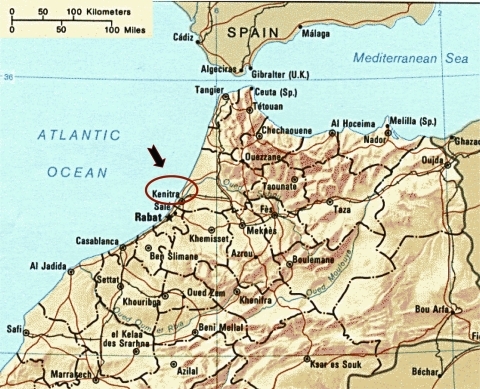
Map showing Kenitra Province, where equine clinical cases occurred in 2003. (adapted from the Internet site http://www.morocco.com/travel/maps03.html)

Equine clinical cases were reported from 3 locations ≈20 to 30 km northeast of Kenitra (34°18´N, 06°30´W), close to the Sebou River delta and the Atlantic Ocean (Figure 1). Irrigation networks are developed in this farming area. In addition, a natural bird reserve, Sidi Boughaba, is located 15 km southeast of Kenitra, along one of the migratory Europe–sub-Saharan routes, where numerous migrating and breeding birds are found.

Virus isolation was performed from a brain biopsy in the BioPharma laboratory in Rabat, Morocco. Brain suspension was injected onto BSR cells. Cytopathic effect was observed 4 days after infection. WNV was identified by immunofluorescence assay and confirmed by reverse transcription–polymerase chain reaction (RT-PCR). The complete WNV genome was sequenced in the National Reference Center for Arboviruses in Lyon, France, after a single passage of the strain (04.05) on Vero E6 cells. Twenty-five overlapping amplicons were amplified and sequenced on both strands (AY701413). The complete sequence of the strain 96-111 isolated during the 1996 Moroccan equine outbreak was also determined (AY701412).

Pairwise alignments of 96-111 and 04.05 sequences using ClustalW1.7 software (11) showed a 98.9% nucleotide identity and a 99.8% amino-acid identity between the 2 Moroccan isolates. Six amino-acid differences were observed between the 2 strains: 1 in the E gene (I732V), 2 in the NS1 gene (V979I and R1079S), 1 in the NS2a gene (H1262Y), and 2 in the NS3 gene (F1551L and A1754T).

Multiple alignments of Moroccan WNV sequences and other WNV sequences available in GenBank database were generated by ClustalW1.7 software. Phylogenetic trees were constructed by using nucleotide alignments, the Jukes Cantor algorithm, and the neighbor-joining method implemented in molecular evolutionary genetics analysis (MEGA) software (12). The robustness of branching patterns was tested by 1,000 bootstrap pseudoreplications.

Comparison of the complete genome sequences showed a high degree of identity between the Moroccan strains and those of the European/Mediterranean/Kenyan cluster. Paired identity at the nucleotide level ranged from 98.2% to 98.9% and from 98.6% to 99% for 04.05 and 96-111 strains, respectively. Paired nucleotide identity with strains of the Israeli/American cluster ranged from 96.2% to 96.3% and 96.5% to 96.6%, respectively. The 5 amino-acid residues characteristic of the European/Mediterranean/Kenyan genotype were conserved in both Moroccan strains, i.e., T416 (E protein), S861 (NS1 protein), I1861 (NS3 protein), V2209 (NS4a protein), and D2522 (NS4b protein).

**Figure 2:**
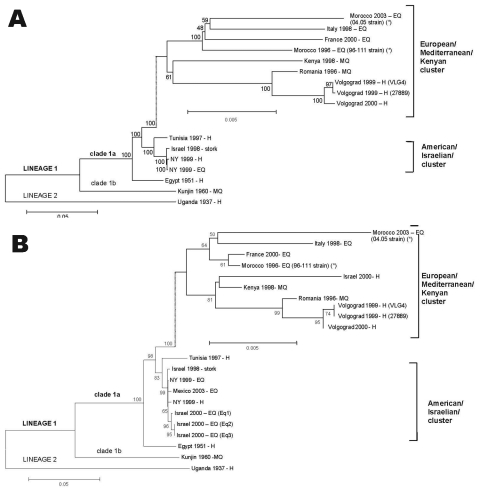
Phylogenetic trees of West Nile Virus (WNV) nucleotide sequences. Phylograms were constructed with the MEGA program, by using the Jukes Cantor algorithm and the neighbor-joining method. The percentage of successful bootstrap replicates is indicated at nodes. The length of branches is proportional to the number of nucleotide changes (% of divergence). The strains sequenced in this study are indicated by asterisks (*). A) Complete nucleotide sequence GenBank accession no. are Italy 1998 (AF404757), France 2000 (AY268132), Kenya 1998 (AY262283), Romania 1996 (AF260969), Volgograd 1999 (VLG4) (AF317203), Volgograd 1999 (27889) (AY277252), Volgograd 2000 (AY278442), Tunisia 1997 (AY268133), Israel 1998 (AF481864), NY 1999 H (AF202541), NY 1999 EQ (AF260967), Egypt 1951 (AF260968), Kunjin 1960 (D00246), Uganda 1937 (M12294). B) Envelope nucleotide sequence Genbank accession no. are Italy 1998 (AF404757), France 2000 (AY268132), Israël 2000 H (AF394217), Kenya 1998 (AY262283), Romania 1996 (AF260969), Volgograd 1999 (VLG4) (AF317203), Volgograd 1999 (27889) (AY277252), Volgograd 2000 (AY278442), Tunisia 1997 (AY268133), Israel 1998 (AF481864), NY 1999 EQ (AF260967), Mexico 2003 (AY426741), Israël 2000 Eq1 (AF380669), Israel 2000 Eq2 (AY052406), Israel 2000 Eq3 (AY052407), NY 1999 H (AF202541), Egypt 1951 (AF260968), Kunjin 1960 (D00246), Uganda 1937 (M12294). EQ: equine; H: human; MQ: mosquito.

On the basis of the complete genome sequences, phylogenetic data showed that both Moroccan strains belonged to the clade 1a of the lineage 1 and clustered with the strains of the European/Mediterranean/Kenyan cluster (Figure 2A). On the basis of the envelope sequences, equine WNV strains isolated in the Mediterranean basin from 1996 to 2003 belonged to 2 distinct clusters, i.e., the European/Mediterranean/Kenyan cluster and the American/Israeli cluster (Figure 2B). The Moroccan equine strains clustered with the Italian and French equine strains. They were more distantly related to the 3 equine strains isolated in Israel in 2000.

## Conclusions

WNV has been circulating in the Mediterranean basin for a long time (1–3); in the western part of the basin, only a few isolates have been obtained and completely sequenced. We report here the isolation and complete genome characterization of 2 WNV strains involved in equine outbreaks in Morocco in 1996 and more recently in 2003.

During the late summer of 2003, an equine outbreak was reported in Morocco. By contrast with the 1996 outbreak (10), the epidemic was restricted geographically and temporally. Climatic and vectorial conditions might have been insufficient to lead to a major transmission of the virus. No young horses were clinically affected, probably because of the structure of the equine population in Kenitra Province, where most horses are bought at the age of 4 or 5 years and the mean age of the equine population is 10 years.

High pairwise nucleotide and amino-acid identity values indicated that the Morocco 1996 and 2003 WNV strains are closely related. Determining if both outbreaks were related to an endemic strain or to distinct introduction events by migratory birds was not possible. Since 1996, WNV-positive serologic results have been found every year in horses with neurologic signs, which suggests endemic circulation of the virus (M. el Harrak, unpub. data.).

On the basis of envelope and complete genome sequences, we demonstrated that both Moroccan strains belonged to the European/Mediterranean/Kenyan cluster previously defined (8). The characterization of 2 new complete WNV genome sequences allowed us to demonstrate the genetic stability of the WNV strains involved in the equine outbreaks reported since 1996 in the western part of the Mediterranean basin. Our data also suggested the existence of 2 subclusters of WNV strains in the European/Mediterranean/Kenyan cluster. One subcluster includes strains isolated in the western Mediterranean basin (France, Italy, Morocco) that have probably been introduced from West Africa. The other subcluster includes strains isolated in the eastern Mediterranean basin (Israel) and southeastern Europe (Romania, Volgograd) that have probably been introduced from East Africa. The molecular epidemiologic features of the strains in the Mediterranean basin appear to be more complex. Since 1997, at least 2 lineages cocirculate in Israel, i.e., the European/Mediterranean/Kenyan lineage and the more recent Israeli/American lineage in birds, equines, and humans (9,13). Strains of the latter genotype were imported in 1999, probably through infected birds or mosquitos, from the Middle East to North America, causing high rates of avian deaths and high rates of illness and deaths in humans and equines. In Israel, the emergence of the Israeli/American genotype has also been associated with avian deaths. Whether the introduction of this genotype is associated with the high rates of illness and death during the 2000 human outbreak is unclear. Five amino-acid residues are known to distinguish the European/Mediterranean/Kenyan and the Israeli/American genotypes (7,8). In the future, testing the role of those specific residues and comparing the biologic properties of strains of both genotypes will be useful, knowing that only Israeli/American strains are responsible for avian deaths (14) and probable increased neurovirulence (15). No viruses of the Israeli/American genotype have been isolated elsewhere in the Mediterranean basin or in Europe.

During the Morocco 2003 outbreak, WNV reemerged in southern France, causing 7 human and 4 equine cases, and in Tunisia, causing approximately 200 human cases in Monastir province (H. Trikki, pers. comm.). No virus isolation or genome amplification was obtained or reported. Knowing the possibility of transmission through blood donations, surveillance of WNV infections must be enhanced in the Mediterranean basin. For the moment, in contrast with the situation in North America, human and equine outbreaks have been restricted geographically and temporally (3). The mechanisms of WNV reintroduction in Europe and in the Mediterranean basin and the cycle of maintenance in infected areas remain to be elucidated. Further studies should focus on competence of mosquito vectors, identifying bird species involved in the cycle of transmission, and the persistence mechanisms of the virus in WNV-endemic areas.
